# Identifying Causal Genes and Dysregulated Pathways in Complex Diseases

**DOI:** 10.1371/journal.pcbi.1001095

**Published:** 2011-03-03

**Authors:** Yoo-Ah Kim, Stefan Wuchty, Teresa M. Przytycka

**Affiliations:** National Center for Biotechnology Information, National Library of Medicine, National Institutes of Health, Bethesda, Maryland, United States of America; Stanford University, United States of America

## Abstract

In complex diseases, various combinations of genomic perturbations often lead to the same phenotype. On a molecular level, combinations of genomic perturbations are assumed to dys-regulate the same cellular pathways. Such a pathway-centric perspective is fundamental to understanding the mechanisms of complex diseases and the identification of potential drug targets. In order to provide an integrated perspective on complex disease mechanisms, we developed a novel computational method to simultaneously identify causal genes and dys-regulated pathways. First, we identified a representative set of genes that are differentially expressed in cancer compared to non-tumor control cases. Assuming that disease-associated gene expression changes are caused by genomic alterations, we determined potential paths from such genomic causes to target genes through a network of molecular interactions. Applying our method to sets of genomic alterations and gene expression profiles of 158 Glioblastoma multiforme (GBM) patients we uncovered candidate causal genes and causal paths that are potentially responsible for the altered expression of disease genes. We discovered a set of putative causal genes that potentially play a role in the disease. Combining an expression Quantitative Trait Loci (eQTL) analysis with pathway information, our approach allowed us not only to identify potential causal genes but also to find intermediate nodes and pathways mediating the information flow between causal and target genes. Our results indicate that different genomic perturbations indeed dys-regulate the same functional pathways, supporting a pathway-centric perspective of cancer. While copy number alterations and gene expression data of glioblastoma patients provided opportunities to test our approach, our method can be applied to any disease system where genetic variations play a fundamental causal role.

## Author Summary

It is now being recognized that complex diseases should be studied from the perspective of dys-regulated pathways and processes rather than individual genes. Indeed, various combinations of molecular perturbations might lead to the same disease. In such cases, responses to these perturbations are expected to converge to common pathways. In addition, signals that are associated with each individual perturbation might be weak, rendering studies of complex diseases particularly challenging. Aiming to provide an integrated perspective on complex disease mechanisms we developed a novel computational method to simultaneously identify causal genes and dys-regulated pathways. Starting with an identification of a disease-associated set of genes and their statistical associations with genomic alterations, we utilized graph-theoretical techniques and combinatorial algorithms to determine potential paths from the genomic causes through a network of molecular interactions. We applied our method to sets of genomic alterations and gene expression profiles of Glioblastoma multiforme (GBM) patients, uncovering candidate causal genes and causal paths that are potentially responsible for the altered expression of disease associated target genes. While copy number alterations and gene expression data of GBM patients provided opportunities to test our approach, our method can be applied to any disease system where genetic alterations play a fundamental causal role, and provides an important step toward the understanding of complex diseases.

## Introduction

Complex diseases are typically caused by combinations of molecular perturbations that might vary strongly in different patients, yet dys-regulate the same component of a cellular system [1]. In recent years, whole-genome gene expression sets have increasingly been used to search for markers, allowing an improved diagnosis of diseases or classification of their subtypes [2], [3], [4], [5], [6], [7], [8]. Several approaches combined expression measurements with various types of direct or indirect pathway information, leading to improved disease classification [9], [10], [11], [12], prioritization of disease associated genes [13], [14], [15] and identification of disease specific dysregulated pathways [16]. Furthermore, considerable efforts towards integrated approaches for uncovering disease causing genes [17], [18] and elucidation of relations between variability in gene expression and genotype [19] have recently been made. In particular, Tu *et al*. developed a random walk approach to infer regulatory pathways [13], [14], [20] in yeast. Suthram et al. [21] further improved this approach by using the analogy between random walks and current flow in electric circuits. Recently, Yeger-Lotem et al. developed a min-cost flow based algorithm, uncovering cellular pathways that are implicated in several neurodegenerative disorders [22].

Studying associations between individual disease genes and genotype alterations allowed us to uncover potential causative factors and affected molecular entities. While previous methods provided valuable insights into the modular nature of diseases by elucidating groups of differentially expressed genes, the flow of information from potential causes to effected genes in the molecular interaction network hasn't been investigated. In this paper, we present a genome-wide approach to simultaneously determine dys-regulated pathways and their putative causes/factors. We utilized gene expression and genomic alteration profiles of 158 glioblastoma multiforme (GBM) patients. We started by selecting a set of differentially expressed target genes, and then identified pathways connecting genes that are located in areas of genomic alterations. Then, we selected target genes, choosing pathways that are likely to explain the expression abnormalities of target genes. Consistent with the general strategy of eQTL analysis, we assumed that expression variations of the target genes are, at least in part, caused by genomic alterations. Specifically, we first used association analysis to identify possible cause-target gene pairs. Then, we modeled the propagation of information from a potentially causal gene to a target gene as the flow of electric current through a network of molecular interactions. To assess the significance of identified pathways we carefully designed a permutation test. Finally, we used a graph-theoretical approach to further narrow down the selected set of putative causal genes. We validated our approach by testing the enrichment of selected causal genes with known GBM/Glioma disease genes and literature searches. We also examined the subnetworks, connecting causal and target genes and identified cancer hub genes and sets of functionally related genes which indicate involvement of specific cellular pathways. Among these pathways we found several expected key players such as EGFR and Insulin Receptor signaling pathways, RAS signaling, as well as a glioma-associated regulation of transforming growth factor-β2 production and SMAD pathway. Importantly, such pathways can be considered as “GO biological process hubs” or “highways”, connecting many different causal genes with their targets. Such an observation supports the hypothesis that many different genomic alterations potentially dys-regulate the same pathways in complex diseases. In addition, we analyzed the global properties of identified associations and found a cluster of causal/disease gene activities on chromosomes that are strongly affected by genomic alterations. Such results allowed us to identify candidate causal genes for prominent signaling and regulation proteins that putatively play a role in GBM. Comparing our method to a basic genome-wide association approach, we demonstrated the increased predictive power of our model.

## Results

### Outline of the Method

**Figure 1:**
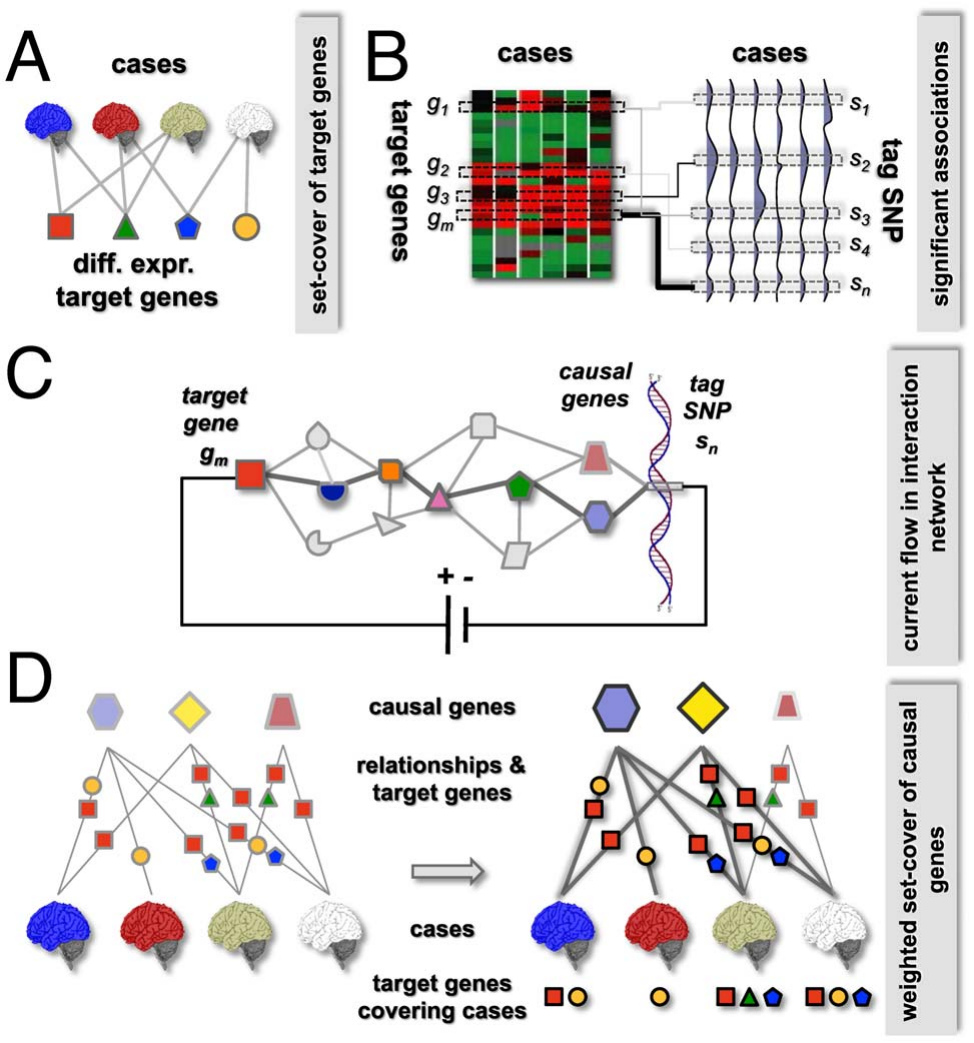
Outline of our method. (**A**) We first selected target genes that were differentially expressed in disease cases, using a multi-set cover approach. (**B**) In the second step, we detected genome-wide associations between gene expression changes of target genes and genomic alterations, allowing us to find potential causal genomic areas. (**C**) In the third step, we determined causal paths from genomic alterations (*i.e.* causal genes) to target genes by modeling and solving a current flow problem through a circuit of molecular interactions. (**D**) To select a final set of causal genes, we designed a weighted multi-set cover algorithm. Constructing a bipartite graph between candidate causal genes and disease cases, we labeled each edge with the associated set of target genes that were affected by the causal gene and were differentially expressed in the corresponding disease case. In the final set-cover, causal genes in boxes covered each disease case with at least two target genes, allowing one exception.

We developed a novel computational method to identify causal genes and associated dys-regulated pathways by an integration of several layers of data, including profiles of gene expression and genomic alterations (Fig. 1). Specifically, we assembled an interaction network, utilizing molecular interaction data such as protein-protein interactions, phosphorylation events and protein-transcription factor interactions. Briefly, our algorithm consists of four main steps (Figures 1A–D): (i) selection of a set of differentially expressed target genes, (ii) identification of possible causal loci of each target gene by an eQTL-analysis, (iii) identification of a set of putative causal genes by determining pathways between causal and target genes through the network of molecular interactions, and (iv) determination of a subset of causal genes that best explain the underlying disease cases. In the following, we present a more detailed description of these four steps. Further details are described in the corresponding sections of Materials & Methods.

### Selecting Target Genes in GBMs

**Figure 2:**
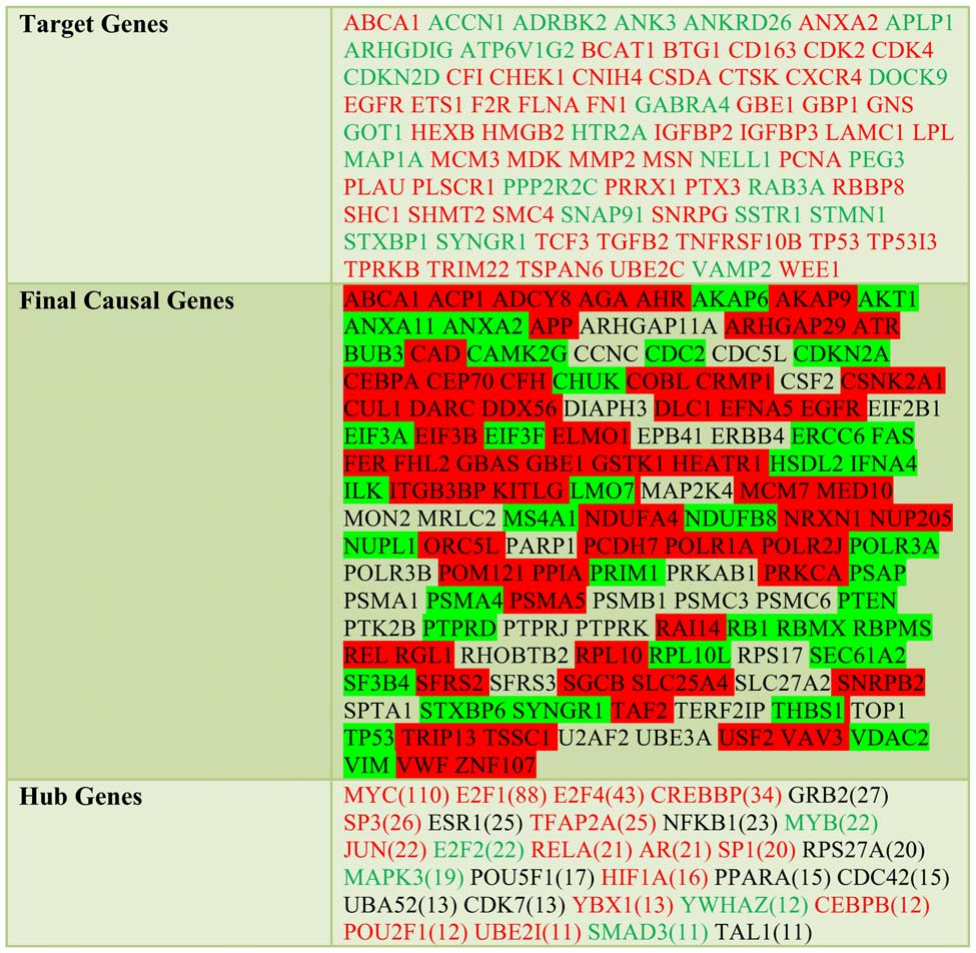
Lists of selected target, causal and hub genes. Target and hub genes that are labeled red were up-regulated while genes labeled green were down-regulated. Causal genes are marked in red (green) if they were found in amplified (deleted) genomic regions. We defined hubs as genes that appeared in more than 10 causal pathways through the interaction network. Numbers in parentheses indicate the genes' actual occurrences.

Since a complex disease may manifest itself differently in patients, we first developed a method that selects a set of genes that are differentially expressed in the disease cases and cover individual patient alterations. To identify such representative genes, we modeled the selection of target genes as a multi-set cover problem (Fig. 1A). Specifically, we determined a set of genes that were differentially expressed in 158 glioblastoma cases compared to 32 non-tumor control cases (see selection of target genes section in Materials & Methods). We defined that a differentially expressed gene *covers* a particular disease case if the gene was differentially expressed in the underlying case. Clearly, genes that cover many cases are expected to represent genes and pathways commonly dys-regulated in the disease. To capture disease heterogeneities we also demanded that each disease case was covered by at least a certain number of target genes, a key parameter of our approach. Intuitively, with very small coverage we can identify only the most commonly differentially expressed genes. By increasing coverage we can capture genes that are specific to smaller subgroups of patients. Thus, we required a certain level of coverage and simultaneously demanded that each gene covers as many cases as possible. To achieve this goal, we formulated the problem as a minimum multi-set cover (see selection of target genes section in Materials & Methods) and solved it using a greedy algorithm. We tested several combinations of coverage and the number of outliers (a second, less prominent parameter of the algorithm) and observed that obtained gene sets strongly overlapped, demonstrating the robustness of our approach (see Text S1 for details of the algorithm and parameter settings). Demanding coverage of 55 and allowing 3 outliers, we selected 74 target genes as presented in Fig. 2 (see Table S1 for an annotated list of target genes).

### Association between Gene Expression and Copy Number Alterations

The goal of this step is to identify an initial set of possible associations between copy number variations and target genes for further analysis (Fig. 1B). Since genomic variations in neighboring regions tend to be highly correlated, we first chose a subset of 911 representative loci (i.e. *tag loci*), significantly lowering computational costs (see eQTL mapping section in Materials and Methods). We observed that the number of genes that a tag locus can harbor varied strongly and found on average 27 genes per tag locus. Applying a standard eQTL approach [19], [23] we performed a linear regression analysis, allowing us to determine genome-wide associations between the expression of target genes and copy number alterations of tag loci. Specifically, we calculated p-values for each gene-locus pair under the null hypothesis that the slope of the linear regression is 0. This way we selected, for further analysis, 3,091 associated gene-locus pairs (p<0.01), amounting to <5% of all 67,414 (911×74) tested pairs. On average, we selected 41 associated tag loci per target gene, while 776 tag loci had at least one target gene (see Text S1 for algorithmic details).

### Candidate Causal Genes in eQTL Regions

The relatively liberal p-value threshold used in the previous step allowed us to retain most of potentially interesting relationships. Although this step filtered out the least promising pairs, a large number of false positives are expected to pass this threshold. Reducing false discovery rate by simply decreasing the p-value threshold would retain extremely well correlated loci-target gene pairs only, therefore missing a large spectrum of potentially interesting pairs. In fact, the correlation between copy number variation in the causal gene and the gene expression of its target gene doesn't have to be strong since such a signal might have been affected by varying degradation rates and posttranslational modifications. Furthermore, genotypic alterations in several loci might lead to the dys-regulation of the same pathway and therefore changing the expression of a target gene in potentially non-additive, epistatic ways. Since each genetically altered region might harbor a large number of genes, we also aimed to identify the most likely causal genes within each region.

In order to account for such effects we utilized protein-protein, protein-DNA and phosphorylation networks (Fig. 1C). Existence of statistically significant paths through an interaction network, connecting putative causal and target genes not only provides additional support for the relationship but also helps to identify genes that participate in propagating the signal. This approach also allows identifying the gene(s) within the altered regions which were most likely the cause of the observed expression changes of the selected target genes (Fig. 1C). Motivated by the results of Suthram et al. [21], we adopted a variant of a circuit flow algorithm and modeled the problem of finding a pathway through an interaction network as current flow in an electric circuit. We defined the conductance of each interaction as a function of the expression correlation of the genes at the endpoints of edges and the target gene. Such a model allows the current to preferentially use interactions that more likely mediate information from a causal to a target gene. Stipulating that only transcription factors can change the expression of genes, we required that a causal path ended with a link between a transcription factor and the target gene. The flow of current from the target to its potential causal genes was computed by solving a system of linear equations, allowing us to find a set of candidate causal genes for each target gene. Importantly, we considered edges corresponding to phosphorylation events and protein-DNA interactions as directed, prompting a computational problem that theoretically can be tackled with a linear programming approach [21]. However, the large size of the underlying human interaction network imposed considerable computational costs, prompting us to develop a heuristic that preserved the directions of such molecular interactions. As a null-model, we utilized a permutation test to estimate the statistical significance of the current flow. After obtaining empirical p-values we selected candidate causal genes for each target gene if the empirical, gene specific p-value was <0.05 (for algorithmic details and parameter settings please see solution of the electric circuit problem section in Materials & Methods and Supplement Text S1). We obtained 1,763 pairs, consisting of 74 target and 701 potential causal genes that included a significant number of GBM and glioma-specific genes (Table 1). Since we identified associated gene-locus pairs with p<0.01 and found target-causal gene pairs with p<0.05, all 1,763 pairs had an estimated nominal p-value <5×10^−4^.

**Table 1:** Functional analysis of genes selected in each step.

	A. Number of Genes	B. AceView (GBM)	C. DAVID (Glioma)
Genome-wide association analysis	16056	0.56 (75)	0.027 (56)
Genome-wide association analysis + Bonferroni correction	1026	0.0029 (12)	None
Circuit flow algorithm	701	0.045 (10)	1.3×10^−10^ (25)
Circuit flow + Bonferroni correction	280	0.17 (4)	1.4×10^−7^(16)
Circuit flow + set cover	128	4.7×10^−4^ (6)	4.6×10^−4^ (8)

(**A**) To determine the statistical significance of selected genes, we counted the number of genes identified in each step of our analysis. (**B**) Utilizing a set of 93 genes that are implicated in GBMs as of Aceview we calculated the statistical significance of the overlap (numbers in parentheses) with a hypergeometric distribution. We found that the significance increased, applying the steps in our approach. (**C**) Calculating p-values with a modified Fisher's exact test, we obtained a similar result for a set of glioma genes as of DAVID as well.

### Final Causal Genes Explaining Disease Cases

While the electric circuit approach reduced the number of putative causal genes significantly, the size of this gene set was still considerably large. In the final step, we applied another filter by considering two approaches – a statistical method and a hypothesis driven optimization approach. In the statistical approach, we accounted for multiple hypothesis testing and used a p-value cut-off of 5×10^−8^, producing 280 candidate causal genes. In the optimization-based approach, we identified relevant causal genes by selecting the set of genes that best explained all disease cases. We defined that a putative causal gene *explains* a disease case if its corresponding tag locus has a copy number alteration and its affected target genes (*i.e.*, genes sending a significant amount of current to the causal gene) were differentially expressed in the underlying disease case. In other words, if a link between a causal gene and a disease case existed, we expected to observe both a genomic alteration of a causal gene and differential expression of its target gene in the same disease case. Since a causal gene may potentially affect one or more target genes, we defined the *weight* of the explanation as the number of such target genes. Therefore, a gene that explained a disease by perturbing a larger number of target genes had a higher weight, increasing the likelihood to be chosen as a final causal gene (Fig. 1D). To choose a set of causal genes explaining all cases except a few outliers with a minimum number of causal genes, we formulated the problem as a variant of the *minimum weighted multi-set cover problem* (please see selecting a final set of causal genes section in Materials & Methods and Text S1 for algorithmic details). Utilizing a greedy algorithm, we determined a set of 128 putative, final causal genes that were involved in 625 causal and target gene pairs. Using a permutation test, we found that the random selection of a gene set of at most this size occurred with p<3.1×10^−4^.

### Validation

In the following, we provide a quantitative validation of the set of putative causal genes, pathway hubs and target genes. Where applicable, we also compared our results to previous approaches. Subsequently, we established the robustness of our method with respect to parameter settings. Finally, we analyzed individual genes and pathways.

To assess the significance of our set of causal genes, we determined the overlap with sets of GBM/glioma specific genes. In particular, AceView [24] provided a list of 93 GBM specific genes. In the first step of the algorithm, we determined associations between copy number variations and expression of target genes, yielding 16,056 associated genes that had a large, but statistically insignificant overlap with the set of glioblastoma specific genes (p<0.56, Table 1). The application of the electric circuit algorithm reduced this set to 701 candidate causal genes with a significant enrichment of 10 GBM specific and 25 Glioma related genes (p<0.05, Table 1). We also checked the advantage of using the current flow approach instead of simply selecting pairs based on more stringent p-value cut-offs. Namely, given our eQTL results, we used a Bonferroni-corrected threshold of 1.5×10^−7^, providing 24 pairs between 4 target genes and 22 loci that harbor a total of 1,026 genes, including 12 GBM relevant genes from AceView (p<0.003, Table 1). However, this approach failed to find any significant associations for most of the target genes. For the 4 target genes, we obtained a rather big set of candidate causal genes, which was not enriched with glioma genes in DAVID.

Next, we focused on the last step of the algorithm. As a result of the current flow step we obtained 1,763 pairs with a nominal p-value <5×10^−4^, involving 701 causal genes. Using the weighted set cover approach, we identified 128 causal genes that harbored 6 GBM relevant genes (Table 1). Specifically, we found that both sets shared CDKN2A, EGFR, ERBB4, PTEN, RB1 and TP53 (p<4.7×10^−4^). Utilizing a set of glioma relevant genes from DAVID database [25], [26], we obtained consistent results (Table 1). In contrast, by Bonferroni-correcting causal-target gene pairs we obtained 280 causal genes, including only 4 GBM related genes according to AceView (p<0.17, Table 1).

To test an alternative approach, we greedily chose loci with smallest p-values until we pooled at least 128 putative causal genes. The obtained set of putative causal genes included only 2 GBM genes (p<0.3), suggesting that the current flow algorithm and the subsequent filtering step with a set-cover allowed us to uncover more cancer relevant genes than the simple association approach.

Focusing on the final set of 128 causal genes, we utilized canonical pathway data from DAVID and found that the final set of 128 causal genes was significantly enriched with glioma, cell cycle genes, p53 signaling pathway and proteasomal genes (p<0.05). In Table 2 we listed the most enriched annotated pathways, their genes and p-values. The complete list of 128 final causal genes is shown in Fig. 2, and an annotated list is provided in Table S2.

**Table 2:** Functional analysis of final causal genes.

	P-value	Genes
**Glioma**	0.008	PRKCA,EGFR,AKT1,CDKN2A,CAMK2G,TP53,RB1,PTEN
**Cell cycle**	0.028	MCM7,CDKN2A,CDC2,TP53,ORC5L,RB1,ATR,BUB3,CUL1
**p53 signaling pathway**	0.030	CDKN2A,CDC2,TP53,ATR,FAS,THBS1,PTEN
**Proteasome**	0.026	PSMA1,PSMC6,PSMB1,PSMC3,PSMA5,PSMA4

Analyzing the enrichment in different functional gene sets provided by DAVID with a modified Fisher's exact test, we found that our final set of 128 causal genes was significantly overlapping with a set of glioma, cell cycle, p53 signaling and proteasome genes.

We also assessed the importance of genes in the paths from putative causal genes to their target genes. As described in identifying dysregulated pathways section in Materials &Methods, we identified causal paths between a target and a causal gene by finding a maximum current path through the network of molecular interactions. In particular, we demanded that the genes in causal paths have significant p-values while the current passing through all genes in the path is maximized (please see identifying dysregulated pathways section in Materials & Methods and also Text S1 for algorithmic details), allowing us to identify 461 genes in 995 interactions. Using a threshold of more than 10 occurrences in causal paths (corresponding to 20% of most frequently appearing genes), we observed the emergence of hubs, genes that appeared in a disproportionally large number of pathways (Fig. 2). Such a set of hubs contained important transcription factors such as MYC and E2F1 and oncogenes such as JUN and RELA and was enriched with genes that appeared in cancer pathways (p<2.2×10^−8^), the cell cycle (p<3.5×10^−6^) and several important signaling pathways from DAVID. While such hub genes were clearly related to cancer, we hardly would have identified them by analyzing differentially expressed genes or copy number alterations alone, demonstrating that the pathway-based approach considerably helped us to uncover these important players.

Utilizing DAVID, we also found that our target gene set was enriched with genes in the cell cycle (p<7.6×10^−4^), p53 signaling pathway (p<9.1×10^−4^), and RB Tumor Suppressor/Checkpoint Signaling in response to DNA damage (p<4.8×10^−3^). Among target genes, we also found up-regulated WEE1, a tyrosine kinase that phosphorylates CDK1 [27], a signaling event that is crucial for the cyclin-dependent passage of various cell cycle checkpoints. Previous reports suggested that overexpression of WEE1 is critical for the viability of some cancer types, and cell lines displaying higher expression levels of WEE1 are sensitive to WEE1 inhibition [28].

**Figure 3:**
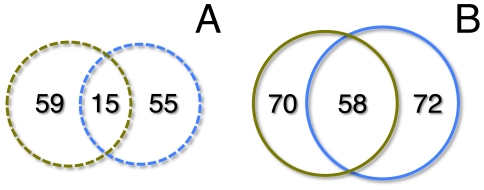
The overlap of two different sets of causal/target genes. In the Venn-diagram in (**A**) we show the overlap of two different sets of target genes. Even though these sets were almost disjoint, we found in (**B**) that the corresponding sets of their causal genes overlapped by up to 45%. Even though the initial sets of target genes were hardly similar, we concluded that our method remarkably compensated this disparity by determining strongly overlapping sets of causal genes.

In an additional test, we eliminated the requirement that the last node on a path leading to a target gene must be a transcription factor. With this change, we selected parameters in our multiset-cover approach to obtain an alternative set with approximately the same number of target genes and we found that it was almost disjoint from our original set of 74 target genes (Fig. 3A). Despite these differences, the final sets of causal genes had a strong overlap (Fig. 3B) of 58 genes that we found in both sets. Such a level of robustness is consistent with a pathway-centric view of complex diseases: different sets of target genes are bundled within dys-regulated pathways that are influenced by specific combinations of causal genes. Even though the two target gene sets looked largely different, both sets include genes that are differentially expressed in the disease cases. In addition, we found that the genes are close relatives in the network: the average distance between the two sets of target genes is 1.7 (p = 1.7×10^−12^), suggesting that the genes were selected from the same dysregulated pathways.

### Chromosomal Analysis of Causal Genes

**Figure 4:**
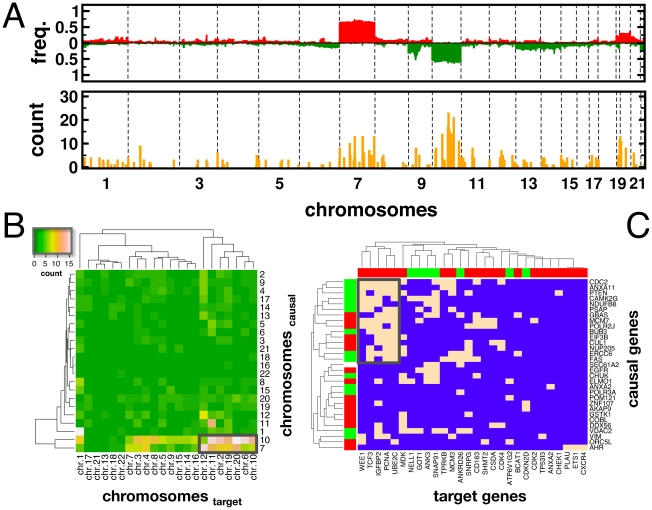
Chromosomal analysis of causal genes. (**A**) In the upper panel, we show the profile of genomic alterations in glioblastomas, where we observed large areas of genomic amplification on chromosome 7 and deletions on chromosome 10. Utilizing predictions of causal genes, we observe that the profile in the lower panel of occurrences (yellow bars) coincide well with the profile of alterations in the upper panel. Focusing on causal genes in the final set-cover (green bars), we recover the initial patterns. In (**B**), we constructed a matrix, showing the number of pairs of target and causal genes on their corresponding chromosomes. We found that causal genes on chromosomes 7 and 10 have numerous links to target genes on chromosomes 2, 3, 6, 10, 11, 12, 19 and 20 (boxed area). (**C**) Focusing on target genes in these chromosomal areas, we marked the presence of a causal path through a molecular interaction network between a target and causal gene as peach in the heat map. While bars indicated the differential expression of the corresponding genes (green: down, red: up), we found a large cluster of up-regulated target genes that were regulated by an array of largely down-regulated causal genes (boxed area).

In Fig. 4A, we show the profile of genomic alterations in GBM where we observed large areas of genomic amplification on chromosome 7 and deletions on chromosome 10 (upper panel), alterations that coincided with the genomic locations of EGFR and PTEN. We located the genomic position of our 128 causal genes and counted the number of corresponding target genes. We largely observed that causal genes on chromosome 7 and 10 were strongly connected to target genes, a pattern that strongly coincided with the signature alterations of GBMs.

Since a target and a causal gene might be located on different chromosomes, we determined the occurrences of such chromosome combinations using all target-causal pairs. Constructing such a matrix (Fig. 4B) we found that strong causal signals emerged from chromosomes 7 and 10. In turn, we observed that target genes fell into three large clusters. In particular, target genes on chromosomes 2, 3, 6, 10, 11, 12, 19 and 20 appeared to have numerous links to causal genes located on chromosomes 7 and 10. Focusing on target and causal genes in these areas, we found a large cluster (box, Fig. 4C) of up-regulated genes that were connected to an array of largely down-regulated causal genes.

### Literature-Based Validation of Individual Causal Genes

In addition, we also looked for literature-based validation of other causal genes. In particular we found RHOBOTB2, a recently discovered tumor suppressor gene [29], in our set of 128 causal genes. We observed that this gene lacked a strong genomic alteration signal, suggesting that our approach was also capable of discovering a subtle causal signature that may have been otherwise missed with a simple disease association analysis. We also found some causal genes with strong genomic alterations that, although not included in AceView nor in DAVID, are well known to be associated with cancer. For example, our final causal gene set included GBAS (for its causal network, see Text S1), a gene that was reported amplified in more than 40% of glioblastomas [30], [31] and CEBPA (enhancer binding protein) that was amplified in about 10% of leukemia cases [32].

### Dysregulated Pathways and Subnetworks

We obtained 128 causal subnetworks from causal genes to their target genes (see identifying dysregulated pathways section in Materials and Methods). For each causal subnetwork, we performed an enrichment analysis of GO-annotated biological processes. Due to the hierarchical structure of GO terms, results included many redundant terms, and general terms tend to have more hits. In Table 3, we listed the most specific GO-annotated biological processes with which more than one subnetworks are enriched. For the full list, see Table S3. In Supplementary Dataset S1 we provided a cytoscape file that allows an interactive exploration of enrichment in the GO hierarchy. The frequently enriched GO processes included several classical cancer-related pathways. For example, 9 causal subnetworks are enriched with epidermal growth factor receptor signaling pathway that has anti-apoptotic properties and may enhance proliferation, invasion, and migration of glioma cells [33], [34], [35]. Similarly, 6 causal-target relationships affected the Insulin signaling pathway. Indeed, recent reports provide an additional evidence for the role of this pathway in glioblastoma [36], supporting the hypothesis that alterations in different genes may dysregulate the same pathways and cause the same disease. Other less frequent pathways were positive regulation of MAP kinase activity, regulation of nitric-oxide synthase activity, estrogen receptor signaling pathway, JAK-STAT cascade and the regulation of transforming growth factor-beta2 production. In particular, transforming growth factor-beta2 (TGFB2) is known to be an important modulator of glioma invasion [37], [38]. Of particular interest is also a related SMAD pathway that occurred in two of our causal subnetworks. While it is debated if this pathway plays a role in TGF β-promoted oncogenesis, a recent study indicated that SMAD-dependent signaling through the induction of PDGF-B has a proliferative and oncogenic role in glioma [39], which is in line with the presence of SMAD genes in our causal subnetworks.

**Table 3:** Enrichment of GO biological processes in causal subnetworks.

GO biological process	#
cell cycle arrest	10
epidermal growth factor receptor signaling pathway	9
negative regulation of cell growth	9
Ras protein signal transduction	9
regulation of sequestering of triglyceride	8
cell proliferation	7
nuclear mRNA splicing, via spliceosome	7
regulation of cholesterol storage	7
nucleotide-excision repair	7
RNA elongation from RNA polymerase II promoter	7
insulin receptor signaling pathway	6
transcription initiation from RNA polymerase II promoter	6
N-terminal peptidyl-lysine acetylation	5
phosphoinositide-mediated signaling	5
positive regulation of lipid storage	4
positive regulation of specific transcription from RNA polymerase II promoter	3
positive regulation of epithelial cell proliferation	3
base-excision repair	2
negative regulation of hydrolase activity	2
gland development	2
positive regulation of MAP kinase activity	2
regulation of nitric-oxide synthase activity	2
estrogen receptor signaling pathway	2
regulation of receptor biosynthetic process	2
response to organic substance	2
JAK-STAT cascade	2
regulation of transforming growth factor-beta2 production	2
G1/S transition of mitotic cell cycle	2
SMAD protein nuclear translocation	2

For each of 128 causal subnets, we determined the enrichment of biological processes as annotated in GO (corrected p-value <0.05, Boferroni corrected). Counting the number of occurrences of each process in the causal subnetworks, we listed *the most specific* GO annotated biological processes that appeared enriched in at least 2 subnetworks.

Testing if these GO-processes were enriched in the set of target genes, we only found an enrichment of a small number of very general, mostly cell-cycle related pathways (see Table S4 for the complete list). Only one term “G1/S transition of mitotic cell cycle” overlapped with the list of most specific terms discovered through the analysis with flow-based causal paths. The lack of specific terms in the GO analysis using target genes was expected since target genes were sampled from multiple dys-regulated pathways, therefore not leading to significant enrichment of specific pathways.

**Figure 5:**
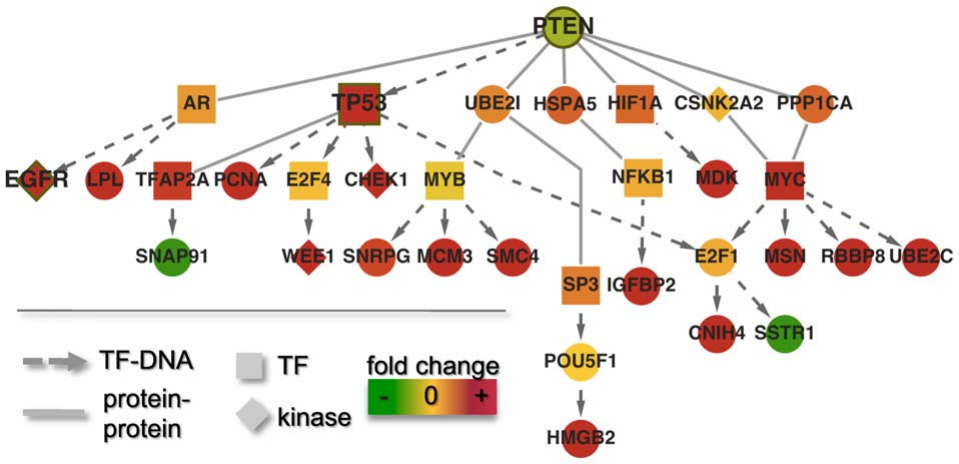
The network of causal paths from PTEN. We observed that PTEN might exert its influence on target genes (the endpoints of each causal path) through prominent transcription factors such as TP53, MYC and MYB.

We took a closer look at paths involving PTEN and EGFR. In Fig. 5, we show a subnet of dysregulated pathways with PTEN as a causal gene. We observed that the influence PTEN might exert on target genes was largely mediated by prominent transcription factors, such as TP53, MYC and MYB. Compared to pathways from DAVID [25], [26], this small network of causal paths was enriched with cell cycle genes (p<0.003) and glioma genes (p<0.02) as well as various types of cancer genes. As their causal roles are indicated in Fig. 4C, we observed that PTEN and CDC2 (see Text S1) might exert their influence on the expression of WEE1 through transcription factors TP53 and E2F4. Since CDC2 codes for CDK1, which is phosphorylated by WEE1 [27] , our results suggest a feedback loop that might be important for cancer.

**Figure 6:**
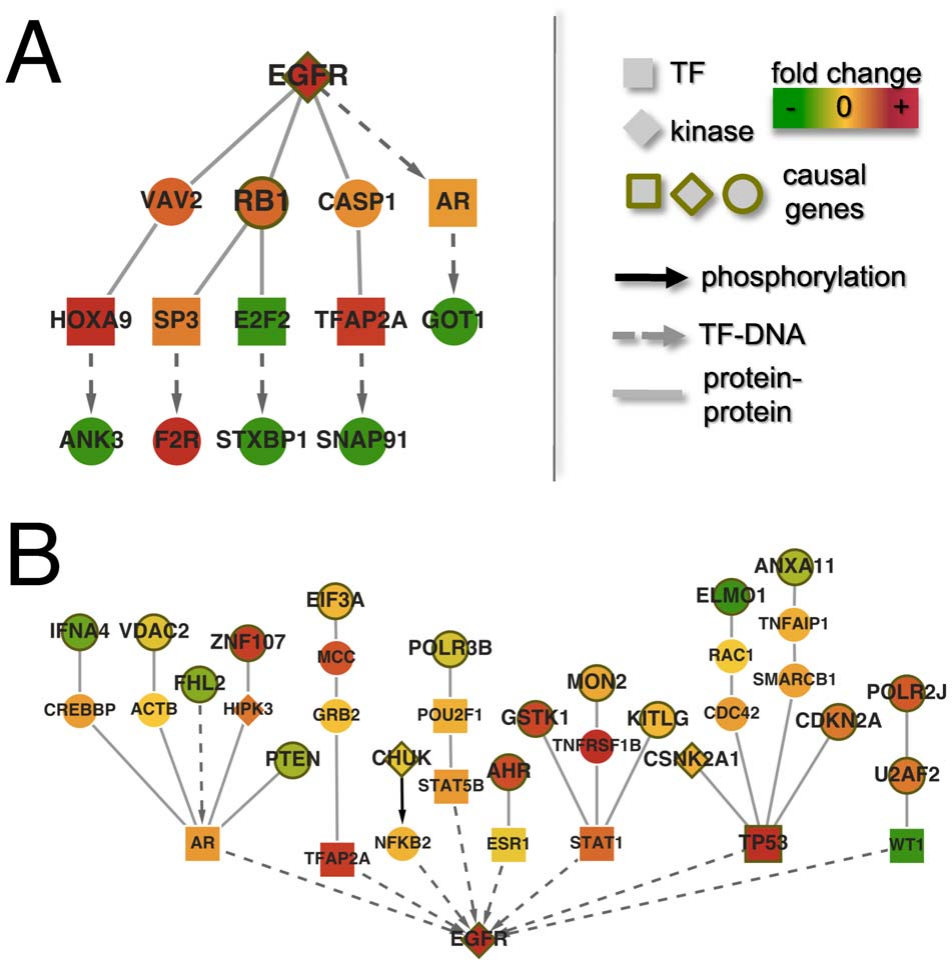
The network of causal paths from and to EGFR. In (**A**) we show a network of causal paths that included EGFR as a causal gene. While this network was rather small, we found a large network of causal paths where EGFR was a target gene in (**B**). Specifically, we observed that EGFR might be influenced by numerous causal genes through prominent transcription factors.

EGFR is highly expressed in disease cases and was selected as both a target and causal gene. The considerable amplifications of chromosome 7 make EGFR a strong candidate for a causal gene. Indeed, we found causal paths that connected EGFR to a few target genes (Fig. 6A). However, we also found a rather large number of causal genes that regulated the expression of EGFR as a target gene (Fig. 6B). Such observations suggest that EGFR might play a dual role as a driver of changed gene expression as well as integrator of causal molecular information from other genomic sites. Indeed, we found numerous disease cases where EGFR was over-expressed without alterations in its genomic location. Instead, we observed that there exist a number of potential causal genes of EGFR with copy number alterations such as ANXA11, CDKN2A, CHUK, PTEN, IFNA4 and ZNF107 among others. Utilizing pathway information from DAVID, we found that the subnet with EGFR as a target gene was highly enriched with glioma genes (p<0.004), the MAPK signaling pathway (p<0.02), and pathways in cancer in general (p<8×10^−8^).

## Discussion

Integrating phenotypic, genomic and interaction data, we introduced a novel approach for the simultaneous identification of causal disease genes and dys-regulated pathways. Such causal genes may include potential drivers of a tumor's emergence as well as potential drug targets. After selecting target genes that covered the underlying disease cases, we determined associations between altered genomic loci and changed expression levels of target genes by a simple eQTL analysis. The key idea of our approach is to combine evidence from association analysis with evidence from pathway analysis. We also demonstrated the power of graph-theoretical approaches in the selection of gene sets and determination of cause-target relationships. Indeed, set cover approaches are increasingly recognized as appropriate tools for selecting disease genes [16], [40], while current flow approaches or equivalent random walk models have been successfully used for modeling of information flow in biological and social networks [41], [42], [43].

Adopting a current flow algorithm, we combined gene expression and molecular interaction data to determine causal paths through interaction networks. This approach allowed for preferential use of network paths supported by expression data, bypassing potential problems of pure topology based methods such as shortest paths that treat all edges equally. Namely, the assignment of resistance to network edges pushed electric current preferentially through nodes that were expression-correlated with the target genes. However, our method also tolerates a fraction of non-correlated nodes, balancing the impact of network connections and a strongly varying degree of gene expression correlation of nodes in the paths.

Current networks of protein interactions, protein-DNA interactions and phosphorylation events are incomplete and noisy. In addition, transcription factors for many genes are unknown, a shortcoming that certainly affected the completeness of our results. However, the problem is alleviated by the fact that cancer is considered as a disease of pathways, suggesting that there exist many ways of selecting a representative set of target genes that represent dys-regulated pathways. Considering a cluster of neighboring genes that participate in the same pathway, any member of the cluster might serve as a target gene to uncover causal genes dys-regulating the underlying pathway. We found that the choice of different target genes provided robust results, diminishing the effects of incomplete data.

We used linear regression for associations to take advantage of its simplicity. To capture the complex relationship of copy number and gene expression more accurately, other non-linear methods can also be considered. However, little is currently known about the precise impact of gene copy number variations on gene expression levels in model organisms, a problem that might even be aggravated by the presence of potential epistatic interactions between loci. In our approach, we alleviated such problems by adopting a relatively liberal p-value cut-off in the initial step of the algorithm. To compensate for this choice, we augmented genome-wide associations with putative paths through a network of molecular interactions. This step allowed us to filter spurious associations and simultaneously uncover other molecules that participate- in the propagation of the perturbation.

Being based on high-throughput interaction data, our approach does not allow us to propose specific molecular mechanisms of signal propagation at this point. Although our method provides an important step forward suggesting potential intermediate nodes for observed associations, uncovered pathways should be considered testable hypotheses rather than ultimate and mechanistic proofs of causal relationships.

The augmentation of associated gene-loci pairs with pathway information resulted in a very powerful strategy, allowing us to not only uncover potential causal genes, but also find intermediate nodes on molecular network paths that mediated information between causal and target genes. Using this method, we also identified functional GO-pathways that mediate many genotype - phenotype associations in GBM. In addition to identifying putative causal genes and dys-regulated functional pathways, our approach provided evidences for the pathway-centric perspective of complex diseases. Firstly, we showed that various genetic perturbations lead to dys-regulation of the same functional pathways. Furthermore, consistent with the hypothesis that genotypic variations dys-regulate whole pathways rather than target individual genes, we found that different sets of target genes sampled from the same pathways lead to uncovering the same causal genotypic variations.

Our method consists of multiple steps of analyses. However, each individual step can be used separately, depending on a specific application. For example, in the first step we selected a set of differentially expressed genes in cancer as target genes. However, this set can be replaced with other user selected set of interest, therefore facilitating targeted studies of particular pathways.

To our best knowledge, our method is the first genome-wide computational approach that reached beyond a simple association analysis. In addition, our method supported genome-wide associations by paths through interaction networks that can, in principle, propagate the information flow from causal genes to target genes. While copy number variation and gene expression data of glioblastoma patients provided an opportunity to test our approach, our method can be applied to any disease system where genetic variations play a fundamental, causal role.

## Materials and Methods

### mRNA Data Treatment

We utilized 158 patient and 32 non-tumor control samples collected from the NCI-sponsored Glioma Molecular Diagnostic Initiative (GMDI) [44], [45] which were profiled using HG-U133 Plus 2.0 arrays. Arrays were normalized at the PM and MM probe level with dChip [44], [46]. Using the average difference model to compute expression values, model-based expression levels were calculated with normalized probe level data. Negative average differences (MM > PM) were set to 0 after log-transforming expression values [44]. Accounting for weak signal intensities, all probesets with more than 10% of zero log-transformed expression values were removed. To represent a gene, we chose the corresponding probeset with the highest mean intensity in the tumor and control samples. Gene expression profiles are available through the Rembrandt database (http://rembrandt.nci.nih.gov/).

### Determination of Copy Number Alterations

All patient and non-tumor control samples were hybridized on the Genechip Human Mapping 100K arrays, and copy numbers were calculated using Affymetrix Copy Number Analysis Tool (CNAT 4). After probe-level normalization and summarization, calculated log_2_-tranformed ratios were used to estimate raw copy numbers. Using a Gaussian approach, raw SNP profiles were smoothed (>500 kb window by default) and segmented using a Hidden Markov Model approach [45], [47], [48]. Genomic alteration profiles are available through the Rembrandt database (http://rembrandt.nci.nih.gov/).

Considering alterations of copy numbers (CN), we defined an amplification if log_2_ CN - 1>0.1 and a deletion if log_2_ CN - 1<−0.1.

### Interaction Network

We utilized human protein-protein interaction data from large-scale high-throughput screens [49], [50], [51] and several interaction databases [52], [53], [54], [55] totaling 93,178 interactions among 11,691 genes. As a reliable source of experimentally confirmed protein-DNA interactions, we used 6,669 interactions between 2,822 transcription factors and structural genes from the TRED database [56]. As for phosphorylation events between kinases and other proteins we used 5,462 interactions between 1,707 human proteins from the networKIN [57], [58] and phosphoELM database [59]. Pooling all interactions we obtained a network of 11,969 human proteins that are connected by 103,966 links.

### Selection of Target Genes

We identified genes that are differentially expressed in the disease cases compared to the non-disease controls in each case. Specifically, we normalized gene expression values as a Z-score, utilizing mean and standard deviation of gene expression values in the non-disease control cases. We considered a gene differentially expressed if the normalized gene expression value of the gene had a *p*-value <0.01 in the given case using a Z-test.

We chose a representative set of target genes by formulating the problem as a minimum multi-set cover. First, we defined a bipartite graph *B(T, S*) between genes *T* and disease cases *S* by adding edges between genes *g* and cases *s* if and only if gene *g* was differentially expressed in case *s.* We constructed a multi-set cover instance *SC*  =  {*B(T, S),α, β*} where *α* represented the number of times that a case needed to be covered, and *β* was the maximum number of outliers. In other words, all but *β* cases needed to be covered at least *α* times in the output cover. The problem to choose a minimum number of genes, satisfying the constraints is NP-hard (*i.e.,* computationally not feasible), prompting us to design a greedy algorithm. The pseudocode of the corresponding algorithm is shown in the Text S1. We demanded that a case needed to be covered at least *α*  = 55 times with a maximum of *β*  = 3 outliers, obtaining 74 target genes.

### eQTL Mapping

We utilized a set of loci *L*  =  {*l_1_, l_2_,…, l_m_*} where each locus *l_i_* was characterized by the corresponding copy number *cn_i,j_* in each case *j*, *CN_i_*  =  {*cn_i,1_, cn_i,2_,…, cn_i,n_*}. Since copy numbers of nearby loci tend to be highly correlated we significantly reduced the number of loci by a local clustering. Specifically, for a potential tag locus *tl_k_*, we greedily accumulated all consecutive loci, ensuring that the Pearson's correlation coefficient of *CN_k_* and *CN_i_* at any locus *l_i_* in the region was *> θ_TL_*  = 0.9. Tag loci and associated regions can be computed in time linear to the number of loci. Note, that adjacent regions may overlap and a gene may belong to more than one region. Given a set of tag loci *TL*  =  {*tl_1_, tl_2_,…, tl_m_*}, we identified candidate causal loci by associating copy number alterations with expression profiles of target genes. Given a set of target genes *TG* and tag loci *TL*, we calculated significant associations by a linear regression between the normalized expression values of gene *tg_i,_ E*(*tg_i_*), and copy numbers of tag locus *tl_j,_ CN*(*tl_j_*). For each target gene *tg_i_*, 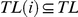
included all tag loci with *p<*0.01. We considered a tag loci *tl_j_* associated with *tg_i_* if *tl_j_* 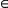 *TL(i).*The pseudocode for selecting tag loci and eQTL mapping is presented in the Text S1.

### Solution of the Electric Circuit Problem

The circuit flow algorithm is based on the well-known analogy between random walks and electronic networks where the amount of current entering a node or an edge in the network is proportional to the expected number of times a random walker will visit the node or edge. Let *G  = * (*N, E*) represent a gene network where *N* is a set of genes and *E* is a set of molecular interactions. Let vector *I*  =  [*I*(*e*) for *e ∈ E*] denote current passing through the edges, and vector *V*  =  [*V*(*n*) for *n ∈ N*] holds variables of voltage at the nodes. For a given tag locus, let *C* be the set of candidate genes located in its genomic region. Vector *X*  =  [*X*[*c*] for *c ∈ C*] denotes the current leaving the candidate genes. For an edge *e  = (u,v)* connecting genes *u* and *v,* we calculated the gene expression correlations *corr(u, tg)* and *corr(v, tg)* between both genes and target gene *tg*. We defined the conductance of edge *e*, *w(e)* as the mean of *corr(u, tg)* and *corr(v, tg)*. As such, we ensured that a single non-correlated node reduced but not completely interrupted the current flow, while a cluster of non-correlated nodes put a considerable resistance to the current flow. Ohm's law is defined as
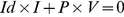
(1)where *Id* is an |*E*|×|*E*| identity matrix, and *O* is a zero matrix. *P* is an |*E*| ×|*N*| matrix and *P*(*e, n*)  =  *w*(*e*) if *n  =  v*, *-w*(*e*) if *n  =  u*, and 0 otherwise. Kirchhoff's current law is
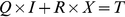
(2)where *Q* is an |*N*| ×|*E*| matrix, and *Q*(*n, e*)  =  1 if *n  =  u*, −1 if *n  =  v*, and 0 otherwise. *R* is an |*N*|×|*C*| matrix where *R*(*n, c*)  =  1 if *n  =  c,* and 0 otherwise. *T* is an |*N*|×1 vector where *T*(*n*)  =  1 if *n* is the target gene *tg*, and 0 otherwise.

Finally, we set the voltage of all genes in *C* to be 0 so that all current flowed into the candidate genes and there is no current flow between candidate genes, defined as
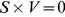
(3)where *S* is a |*C*|×|*N*| matrix and *S*(*c, n*)  = 1 if *n  =  c,* and 0 otherwise.

The set-up of such a linear system implicitly considered all interactions undirected and stipulated that each interaction can have a regulatory effect on the expression of a target gene. In order to obtain more biologically meaningful results, we demanded that direct regulation activity on the expression of target genes is mediated by transcription factors. Therefore, we determined paths where target genes interacted with transcription factors only. In addition, we also accounted for directions of protein-DNA interactions and phosphorylation events. Since linear programming approaches to solve such a directed model [21] required extreme computational resources, we implemented a simple heuristic: after solving the linear system, we removed edges that were used in the wrong direction. We repeated this procedure until only a small number of directed edges were used in the wrong direction (see Text S1 for details). We chose a threshold of 100, which was approximately 0.1% of the total number of edges and found that this heuristic provided a reasonable approximation to the linear programming approach.

### Empirical P-Values

Since the number of genes located in each region varied from 0 to several hundreds, the amount of current that flows to genes cannot be compared directly among different loci to prioritize genes. Given the results of the circuit flow algorithm, an empirical p-value for each pair of a target and a causal gene was estimated, utilizing 30 random networks. Random networks were generated by swapping edges while preserving node degrees to avoid potential biases toward hub nodes. Assuming that each edge had a unit conductance, we ran the circuit flow algorithm in each random network for the same set of genes and computed the amount of current flowing into each gene located in the tag locus. A normal distribution was fitted to the current values in the random networks, and empirical p-values were computed using a Z-test.

For each locus and a set of genes in the associated region, we only considered genes receiving current of at least 70% of the maximum current among all genes in the region. Utilizing the permutation method, we selected candidate causal genes for each target gene if the empirical, gene specific p<0.05. On average, we found a total of 701 causal genes for all 74 target genes (for details of parameter settings, please see Text S1).

### Identifying Dysregulated Pathways

Let *region*(*cg*) be the region that contains a causal gene *cg*. Recall that regions may overlap, and therefore a gene can be part of more than one region. Let *region_max_*(*cg, tg*) and *tl_max_*(*cg, tg*) be the region and tag locus that harbored causal gene *cg* and have the most significant p-value among all the current flow solutions from a target gene *tg* to regions in *region*(*cg*). Utilizing a current flow solution *Sol*(*tg, tl_max_*(*cg, tg*)) from *tg* to *tl_max_*(*cg, tg*), we first removed any nodes with empirical p-value >0.05 from the network. Subsequently, we determined a maximum current path from *tg* to *cg* which was defined as a simple path *P* (*tg, cg*)  =  (*tg, g_1_, g_2_,…, cg*) such that 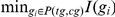 was maximized where *I*(*g_i_*) was the total current passing through the gene *g_i_* (please see Text S1 for algorithmic details). We computed a path for each pair of a final causal gene and a target gene affected by the causal gene.

### Selecting a Final Set of Causal Genes

One of our primary goals was to identify a set of causal genes that explains (almost) all disease cases. Given a set of candidate causal genes and their corresponding copy number variations we identified a subset of common causal genes that explains the disease cases. Specifically, a causal gene *cg_k_* explains a case *s_i_* if (i) the tag locus including the gene has copy number alterations in case *s_i_* and (ii) there exists a nonempty set of target gene(s), *TG(cg_k_, s_i_),* which are affected by *cg_k_* (i.e., with P<0.05) and differentially expressed in case *s_i_*. The weight between a causal gene and a case, *w(k,j)* is defined as *w(k,j)  = * |*TG(cg_k_, s_i_)*|.

A weighted bipartite graph *WB(C, S*) between a set of candidate causal genes *C* and disease cases *S* can be constructed by adding edges between gene *cg_k_* and case *s_i_* if and only if gene *cg_k_* explains a case *s_i_*. For a subset of candidate causal genes *C_0_* and a case *s,* let *W*(*C_0_, s*) be the total number of target genes covering *s* by the genes in C_0_, 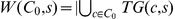. We considered a case as explained if the total weight covering the case exceeds a certain threshold. As in the preprocessing in the first step, we wanted to explain all cases (allowing a few outliers) with minimum number of causal genes (Fig. 1D). The problem can be formulated as a variant of *minimum weighted multi-set cover problem*. Consider an instance *WSC*  =  {*WB(C, S), γ, δ*} where *WB(C, S)* is a weighted bipartite graph between causal genes *C* and cases *S*. We wanted to choose a subset of genes *C'* from *C* such that for each case *s* except *δ* cases, *W*(*C', s*) *≥ γ.* Since a very simple version of the multi-set cover problem (unweighted without outliers) is NP-hard, we designed an algorithm, using a greedy approach to choose a subset of causal genes. Repeatedly, we computed the total weight that can be covered by choosing a gene and selected a gene with maximum additional total weight until the constraints are satisfied (See Text S1 for algorithmic details). Recall that target genes were chosen so that each disease case (except 3 cases) had at least 55 target genes in the first step. As some target genes may not cover the same disease case due to the stricter definition in this step, we found that *δ* = 21 disease cases had less than 50 target genes covering the cases. Therefore, we required an accumulated weight between the set of causal genes and cases *W*(*C', s*) *≥ γ* = 50 in all but *δ* = 21 cases and selected 128 final causal genes.

### Computational Costs

The computationally most expensive component in our algorithm was the circuit flow algorithm. Due to the large size of the human molecular interaction network and the large number of potential causal loci per target gene, the approach required significant computational resources to find a solution to the circuit flow problem and calculate empirical p-values using a permutation method. On average, it took approximately 60-80 hours per target gene to compute solutions for all associated loci (including permutation tests). We used the computing cluster at the NCBI for our computations, allowing us to run several dozens of computations in parallel. In addition, we adapted various optimization techniques to expedite the procedure [60].

## Supporting Information

Dataset S1 Cytoscape file encoding with GO hierarchy of dys-regulated GO processes.
(0.03 MB ZIP)

Table S1 List of selected target genes.
(0.03 MB XLS)

Table S2 List of 128 causal genes.
(0.04 MB XLS)

Table S3 List of enriched GO biological processes in causal subnetworks.
(0.03 MB XLS)

Table S4 List of enriched GO biological processes in target genes.
(0.03 MB XLS)

Text S1 Additional analysis.
(1.40 MB DOC)
