# Supplementary material for: Identifying Causal Genes and Dysregulated Pathways in Complex Diseases
Source: PLoS Comput Biol. 2011 Mar 3;7(3):e1001095. doi: 10.1371/journal.pcbi.1001095 (PMC3048384; doi:10.1371/journal.pcbi.1001095)
Supplement: Text S1 — Additional analysis. (1.40 MB DOC) [file pcbi.1001095.s006.doc]

Indentifying Causal Genes and Dysregulated Pathways in Complex Diseases – Supplementary Material

Yoo-Ah Kim^1^, Stefan Wuchty ^1^, Teresa M. Przytycka ^1^

^1^National Center for Biotechnology Information, NLM, NIH, 8600 Rockville Pike, Building 38A, Bethesda, MD 20894

1. **Pseudocodes**

**Algorithm 1: Pseudocode for selecting target genes.**

1. Construct a bipartite graph *B(T, S*) of genes *T* and disease cases *S* by adding edges between gene *g* and case *s* if gene *g* is differentially expressed in case *s.* Let *S(g*) denote the set of cases to which gene *g* has edges*.*
2. Create a multi-set cover instance *SC* = {*B(T, S) α.β*}
3. *U* = a set of cases covered less than *α*.
4. *TG* = a set of selected genes
5. Repeat the following until |*U*| ≤ *β*:
6. Select a gene $g\notin TG$ with maximum |*U* ∩ *S*(*g*)|
7. Include the selected gene in *TG*
8. Update *U*

**Algorithm 2: Pseudocode of eQTL mapping.**

1. For each chromsome *chr*, let *L_chr_* be the set of loci on the chromosome, sorted in increasing order of their genomic locations.
2. *tl = L_chr_[0]* \\ the first locus
3. Add *tl* to *TL* \\ *TL …* set of TAG loci
4. Consider loci *i* in sorted order:
   1. If *corr (tl, L_chr_[i]) ≤ θ_TL_*: (*corr (x,y)* … Pearson’s correlation coefficient)
      1. *right(tl) = L_chr_[i-1]* \\ set the right boundary of the old TAG locus
      2. *tl = L_chr_[i]* and *tl→TL* [\\ select](../../../../%5C%5Cselect) a new TAG locus
      3. Consider loci in reverse sorted order starting from *j = i – 1*
      4. if *corr(tl, L_chr_[j]) ≤ θ_TL_*:

*left (tl) = L_chr_[j+1]* \\ set the left boundary of the new tag

Go to 4.a

1. For each target gene d*g_i_*:
   1. *TL(i) = []* \\ set of target loci associated with disease gene *i*
   2. For each tag locus *tl_j_:*
      1. Run linear regression between *E(dg_i_)* and *CN(tl_j_)* and compute *p*-value
      2. If p < *θ_eqtl_*:
         1. *tl_j_ → TL(i)*

**Algorithm 3: Pseudocode for selecting candidate causal genes.**

1. For each disease gene *dg_i_*,
   1. *CG*(*dg_i_*)🡨
   2. For each tag locus *tl_j_  TL*(*i*) and associated region *R*(*tl_j_*)
      1. Compute *C*(*tl_j_*), a set of genes located in *R*(*tl_j_*)
      2. Repeat the following:

Construct an electric circuit *G*= {*N, E*}

Compute current *I(g)* to each gene in *C*(*tl_j_*)

If |{*e* in *E*| *e* in reverse direction}| < *θ_r_* :

Go to 1.a.iii

Else:

Remove the edges and repeat 1.a.ii

- - 1. Compute current in random networks and p-values

**Algorithm 4: Finding Dysregulated Pathway from a causal gene *c* to target gene *d.***

1. *r_max_*(*c, d*) 🡨 the region in *r*(*c*) for which *c* has the most significant p-value where *r(c)* is the regions that contain a causal gene *c*)
2. *tl_max_*(*c, d*) 🡨 the corresponding tag locus
3. *G’* 🡨 subgraph of *G* consisting of nodes with p-value > 0.05 in *Sol*(*d, tl_max_*(*c, d*))
4. For each gene g_i_ in *G’*:
   1. *I*(*g_i_*) 🡨 the total current passing through the gene *g_i_* in *Sol*(*d, tl_max_*(*c, d*))
5. *P_max_*(*d, c*) 🡨 paths from *d* to *c* with max *_p∈P_*(*_d, c_*) (min_g_*_i_* _in_ *_p_* *I*(*g_i_*))
6. Choose the shortest path in *P_max_*(*d, c*)

**Algorithm 5: Pseudocode for the selection of final causal genes.**

1. Create a weighted multi-set cover instance *WSC* = {*B, γ, δ*}
2. *U* = a set of cases covered less than* γ.*
3. *MCG* = a set of selected causal genes
4. Repeat the following until |*U*| ≤ *δ*
   1. Select a gene with maximum
   2. Include the selected gene in *MCG*
   3. Update *U*

**2. Parameter settings**

- 1. **Selecting target genes**
     1. Choosing a *p*-value cutoff

We ran the set cover algorithm to select target genes with different p-value thresholds for determining if a gene is differentially expressed for a given case. In Table S5, we listed the sizes of the target gene sets and the overlap with the set presented in the main text. The results in the shaded row are used in the paper. Overall the resulting target gene sets strongly overlap with the current target set by approximately 75-80%, showing that the set cover algorithm is robust to different thresholds.

**Table S5:** Target genes selected with different p-values.

| threshold of p-value for differential expression | set size | overlap |
| --- | --- | --- |
| 0.005 | 94 | 71 |
| 0.01 | 74 | 74 |
| 0.03 | 70 | 56 |
| 0.05 | 67 | 52 |

- - 1. Choosing *α/β*

In the set cover algorithm, we minimize the number of selected genes so that the most prominent genes covering a large set of disease cases are selected. As shown in Fig. S1A, we observed that the number of target genes significantly decreases with *β* = 3 compared to the case where we do not accept any outliers *β* = 0. Increasing the number of outliers to *β* = 6 we find a similar result to *β* = 3.

We have chosen *α* = 55 as the rate of increase slightly goes up after 60. However, we found that the results are not very sensitive to the choice of *α* and *β* as long as the set provides a reasonable cover for disease cases as shown in Figs. S1B-D. In the figures, we showed the size of the target and causal gene sets for each case. We also computed the intersections of the sets with the results presented in the main text (*α* = 55 and *β* = 3) and showed the sizes in the plots. While having more target genes may potentially help find more causal genes, it also requires more computational resources and may increase the chances of selecting false positives.


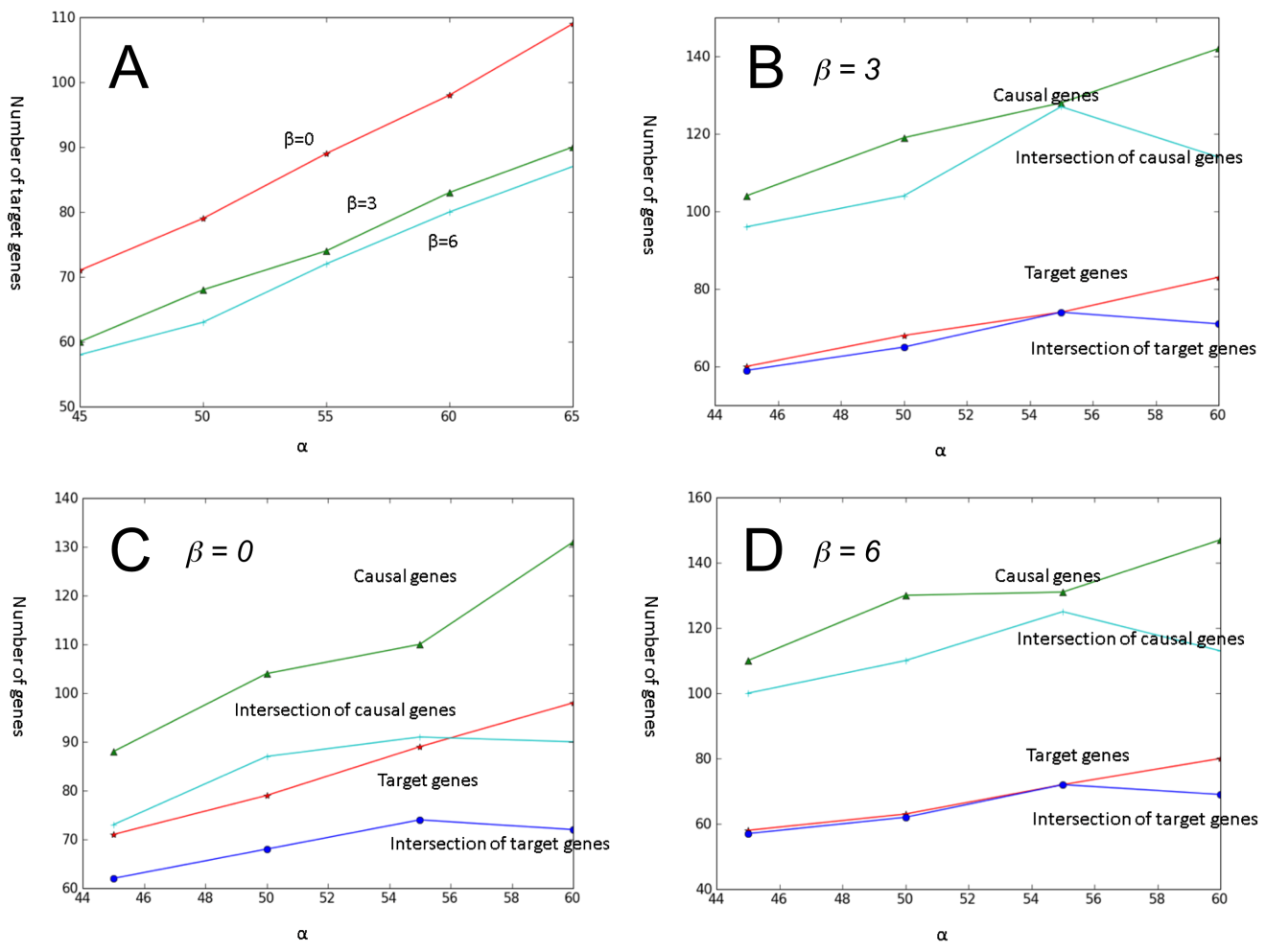


**Figure S1:** **(A)** Number of target genes selected with different *α* and *β*. **(B)** Number of causal and target genes selected and the size of overlap with the current set with different *α* when *β* = 3. **(C)** Number of causal and target genes selected and the size of overlap with the current set with different *α* when *β* = 0. **(D)** Number of causal and target genes selected and the size of overlap with the current set with different *α* when *β* = 6.

- 1. **eQTL mapping**
     1. Choosing a p-value cutoff

We tried two different p-value thresholds for associations and compared the final causal genes (Table S6). If we restrict associated pairs to p-value < 0.005 we find a lower number of pairs (2,071 compared to 3,091) but a larger set of causal genes. The results in the shadowed row are presented in the paper. 81 genes (~ 63%) overlap.

**Table S6: Causal genes selected with different eQTL p-values.**

| eQTL threshold | causal gene set size | overlap |  | GBM related genes |
| --- | --- | --- | --- | --- |
| 0.005 | 157 | 81 |  | CDKN2A ERBB4 RB1 PTEN |
| 0.01 | 128 | 128 |  | CDKN2A EGFR ERBB4 RB1 PTEN TP53 |

- 1. **Circuit Flow algorithm**
     1. Number of reversely used edges

To comply with the directions of edges, we run current flow algorithms repeatedly and remove edges that have been used in the wrong direction in each iteration until the number of wrong edges drops below a certain threshold. We found that most of edges are removed in the first iteration (Fig. S2). Even though we used a stricter threshold (less than 100 edges) which requires 4-5 iterations for most instances, we found that the same final causal genes were selected when we used the solutions obtained after 3 iterations.


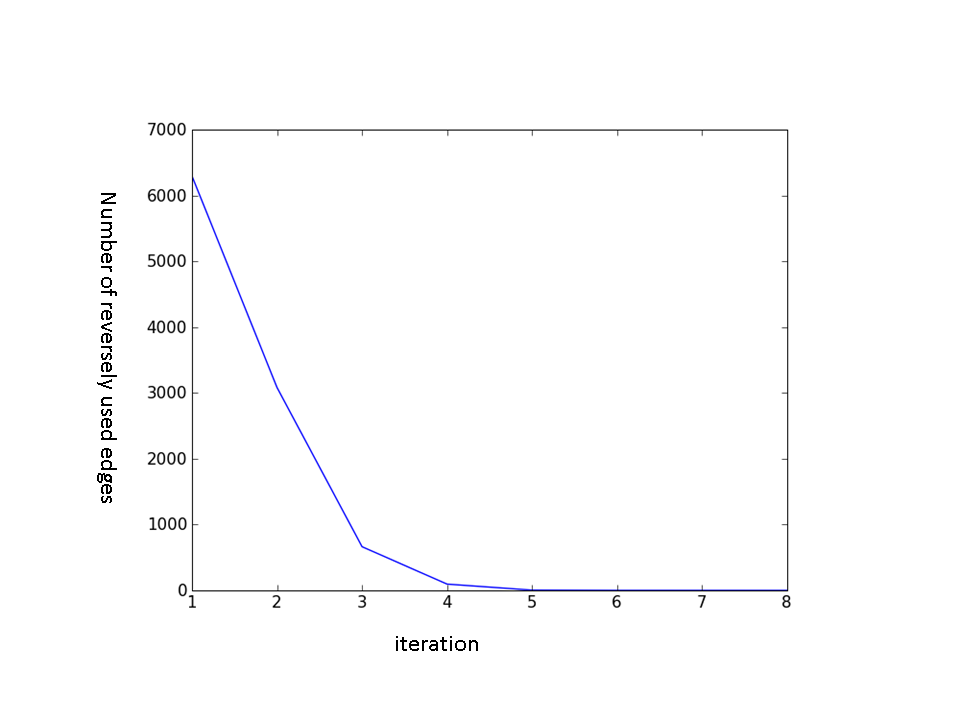


**Figure S2.** For each iteration of the current flow algorithm, we show the average of the number of reversely used edges over all instances.

- - 1. Minimum amount of current

To eliminate genes receiving negligible current, we compared the current amount of each gene with the maximum amount among all the genes in the same tag locus region and considered only the one with the amount above a certain threshold. The overlaps are computed compared with the current set (with 70% of maximum current). The overlap is more than 75% for all cases (Table S7).

**Table S7:** Causal genes selected with different current amount thresholds.

| threshold of current amount | set size | overlap |  | GBM relevant genes |
| --- | --- | --- | --- | --- |
| 0.7 | 128 | 128 |  | CDKN2A EGFR ERBB4 RB1 PTEN TP53 |
| 0.5 | 128 | 114 |  | CDKN2A CTSB EGFR ERBB4 PTEN RB1 |
| 0.3 | 123 | 93 |  | CDKN2A CTSB EGFR ERBB4 PTEN RB1 |

- - 1. Choosing a p-value cutoff

We computed p-values for each gene using the permutation method and used different p-values to decide which target genes are significantly affected by causal gene. The results obtained with p-value of 0.05 are presented in the main text (shaded row, Table S8). In comparison, sets obtained with different threshold have an overlap of >80%.

**Table S8:** Causal genes selected with different p-value thresholds.

| threshold of p-value | set size | overlap |  | GBM relevant genes |
| --- | --- | --- | --- | --- |
| 0.01 | 136 | 109 |  | CDKN2A EGFR ERBB4 PRKDC PTEN RB1 TP53 |
| 0.03 | 134 | 122 |  | CDKN2A EGFR ERBB4 PTEN RB1 TP53 |
| 0.05 | 128 | 128 |  | CDKN2A EGFR ERBB4 RB1 PTEN TP53 |

1. **Additional Materials for Dysregulated Pathways**

**
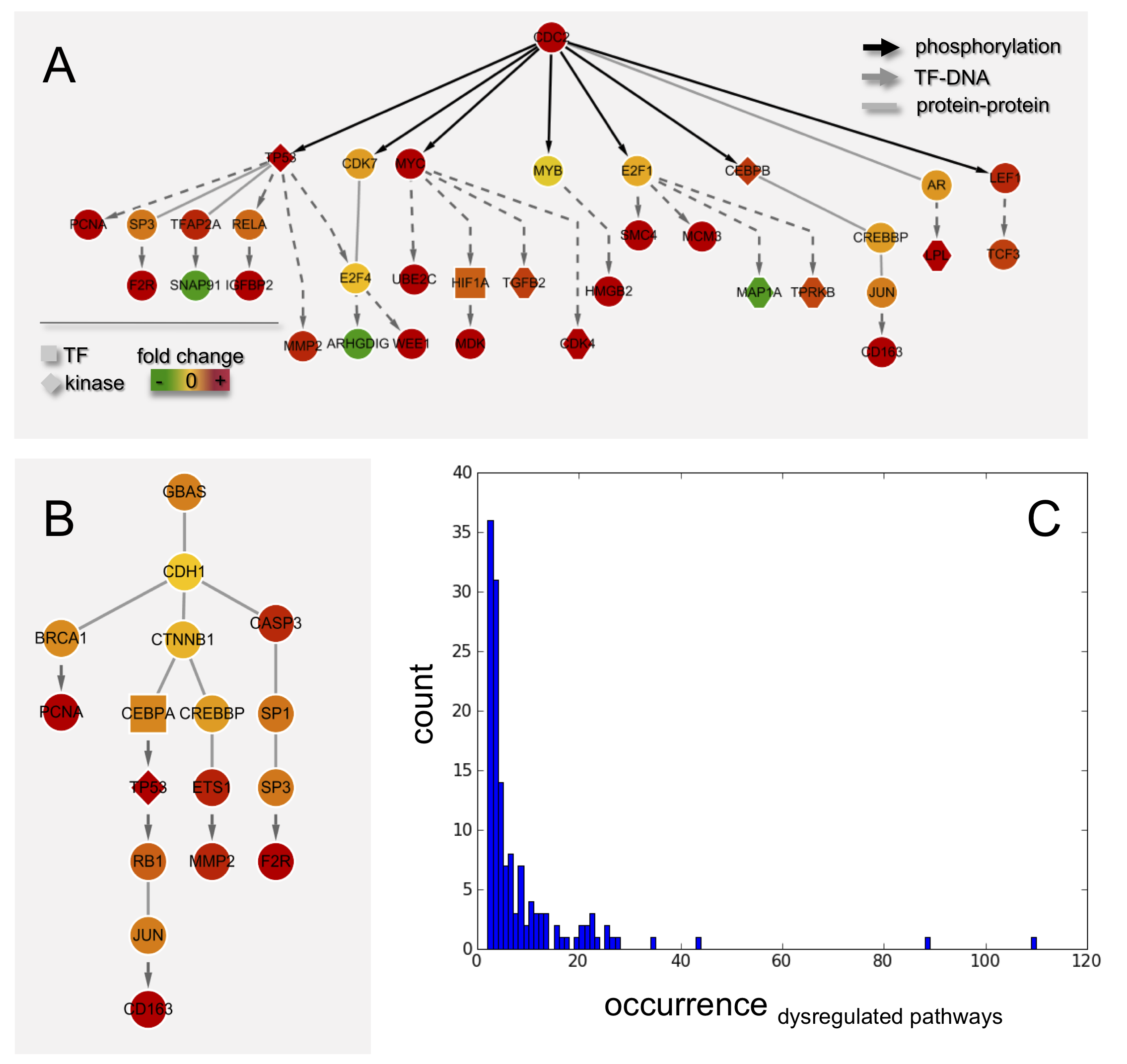
**

**Figure S3: (A)** Dysregulated pathways from causal gene CDC2. Genes in this larger network were significantly enriched in the cell cycle, pathways in cancer, chronic myeloid leukemia, prostate, pancreatic, bladder, colorectal and lung cancers (P < 0.01). **(B)** Dysregulated pathways from causal gene GBAS. Genes that appear in this network are enriched in bladder cancer and cancer pathways in general (P < 0.01). **(C)** Pooling all genes that appear in dys-regulated pathways as presented in the main text, we found that a small number of genes appeared in many causal paths. The hub genes were significantly enriched in cancer pathways, chronic and acute myeloid leukemia, prostate and pancreatic cancers, cell cycle, neurotrophin signaling pathway, renal cell carcinoma, TGF signaling and T-cell receptor signaling pathways (P < 0.001).

1. **GO Enrichment Analysis**

Figure S4 shows a hierchical view of GO enrichment analysis. Since Gene ontology has hierarchical properties, we could generate a DAG (directed acyclic graph) using all enriched GO biological processes. Some GO terms of particular interest are marked in the figure with their locations in the DAG. We also included a table with the list of enriched GO biological processes as Supplementary table S3 and cytoscape file(Supplementary Dataset S1) where one can examine level of enrichment of all GO pathways independently on their specificity .

**
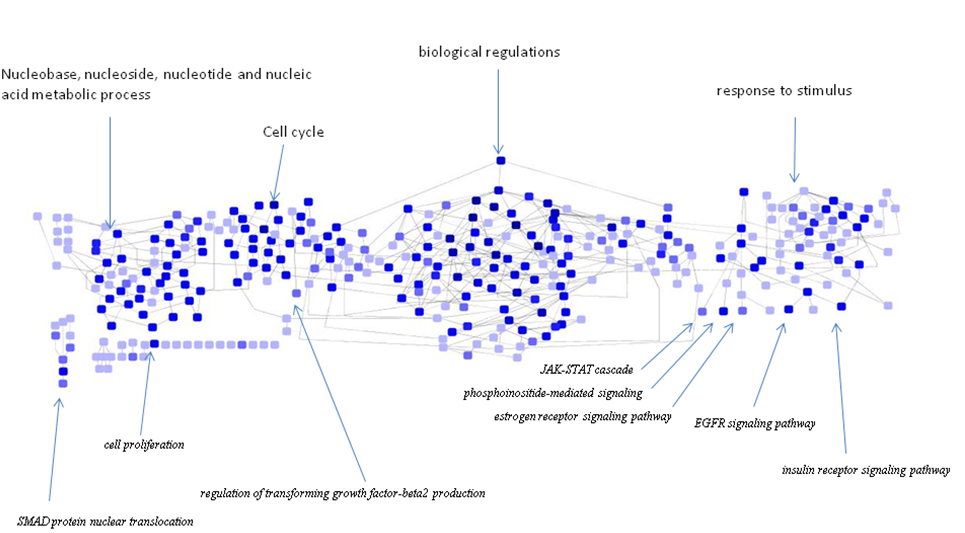
**

**Figure S4:** Hierarchical View of GO biological processes with which the causal subnetworks are enriched. Each node in the graph corresponds to a GO term and the darker color represents the term is enriched in more casuals subnetworks. Terminal nodes are the most specific GO terms and parents nodes are more general than child nodes. The most specific (bottom) nodes are listed in Table S3 and the whole structure can be explored with cytoscape using supplementary data file Dataset S1.
